# Identification of parameters for electronic distance examinations

**DOI:** 10.3389/fvets.2024.1385681

**Published:** 2024-06-19

**Authors:** Robin Richter, Andrea Tipold, Elisabeth Schaper

**Affiliations:** ^1^Centre for E-Learning, Didactics and Educational Research (ZELDA), University of Veterinary Medicine Hannover, Foundation, Hanover, Germany; ^2^Clinic for Small Animals, Neurology, University of Veterinary Medicine Hannover, Foundation, Hanover, Germany

**Keywords:** E-assessment, veterinary education, examinations, open-book, item formats, log data, response time

## Abstract

**Introduction:**

This study investigates the log data and response behavior from invigilated in-person electronic timed exams at the University of Veterinary Medicine Hannover, Foundation, Germany. The primary focus is on understanding how various factors influence the time needed per exam item, including item format, item difficulty, item discrimination and character count. The aim was to use these results to derive recommendations for designing timed online distance examinations, an examination format that has become increasingly important in recent years.

**Methods:**

Data from 216,625 log entries of five electronic exams, taken by a total of 1,241 veterinary medicine students in 2021 and 2022, were analyzed. Various statistical methods were employed to assess the correlations between the recorded parameters.

**Results:**

The analysis revealed that different item formats require varying amounts of time. For instance, image-based question formats and Kprim necessitated more than 60 s per item, whereas one-best-answer multiple-choice questions (MCQs) and individual Key Feature items were effectively completed in less than 60 s. Furthermore, there was a positive correlation between character count and response time, suggesting that longer items require more time. A negative correlation could be verified for the parameters “difficulty” and “discrimination index” towards response time, indicating that more challenging items and those that are less able to differentiate between high- and low-performing students take longer to answer.

**Conclusion:**

The findings highlight the need for careful consideration of the ratio of item formats when defining time limits for exams. Regarding exam design, the literature mentions that time pressure is a critical factor, since it can negatively impact students’ exam performance and some students, such as those with disabilities, are particularly disadvantaged. Therefore, this study emphasizes finding the right time limits to provide sufficient time for answering questions and reducing time pressure. In the context of unsupervised online exams, the findings of this study support previous recommendations that implementation of a stringent time limit might be a useful strategy to reduce cheating.

## 1 Introduction

The digitalization of university teaching has been an ongoing process for decades, which was significantly accelerated and promoted by the COVID-19 pandemic and the associated infection prevention measures (1–5). This was also the case for digitalization measures introduced in veterinary medical education (4–6). One of the most challenging aspects during the pandemic was the issue of conducting examinations. In the light of the infection prevention measures, the formats of traditional in-person written exams and Objective Structured Clinical Examinations were no longer seen as practical for examinations involving large cohorts (4, 7). Distance examinations became the focus as a solution to this challenge.

However, especially in the area of digital distance examinations, there are numerous hurdles in the context of examination regulations and data protection law (3, 7, 8). Particularly concerning online proctoring, which is meant to ensure constant monitoring of the examinees’ identity, protection against attempted cheating, and prevention of the usage of unauthorized tools, many of the currently available technical solutions and tools must be rejected due to European General Data Regulation (GDPR) requirements (8). As a result, the concept of open-book examinations is the main focus of exam design, since in this examination format, apart from direct exchange between candidates, the examinees are allowed to use any form of resources to solve the tasks. Consequently, the need for continuous monitoring to guard against cheating attempts using external sources is limited, and hence the open-book format alleviates the challenging implementation of online proctoring for distance examinations (9).

At the University of Veterinary Medicine Hannover, Foundation (TiHo), Hannover, Germany, electronic examinations have been conducted since 2008 (10), which means that digital performance assessments are already established in both a didactic and technical sense. In the light of the pandemic, the potential of online open-book distance examinations for future examination procedures needed to be assessed. This entailed a review of existing formats and the identification of suitable evaluation parameters. The aim of this study was to evaluate log data from electronic examinations and the examinees’ response selection behavior to determine whether recommendations for the design of online distance examinations can be derived from these data.

## 2 Materials and methods

### 2.1 Selection of data sets

Log data and response selection behavior were examined. A total of 216,625 log records from a cohort of 1,241 students were analyzed, including 225 participants who took multiple exams. Data were derived from one exam from each of five different departments. Examinations from the years 2021 and 2022 were used, with an average participation of 248 individuals per exam (range: 234–282). Care was taken in the selection process to ensure that the summative exams were from various stages of the curriculum, the allocated time per item was varied, and the exams exhibited good characteristic values (Difficulty, Cronbach’s α). The five exams were invigilated electronic state examinations conducted in-person using the Q-Examiner^®^ software (IQuL GmbH, Bergisch Gladbach, Germany); additional details about the exams are provided in Table 1.

**Table 1:** Details on the examinations selected for the analysis of log data sets, including information on the characteristic values of the exams (Cronbach’s α, overall exam difficulty) and item analysis values (Difficulty, Discrimination index).

Subjects		A	B	C	D	E
Section		Clinical sciences	Clinical sciences	Clinical sciences	Veterinary public health	Basic sciences
Number of items		73	90	73	50	60
Exam time (in minutes)		90	120	90	100	90
Cronbach’s α		0.79	0.81	0.81	0.76	0.86
Overall exam difficulty (%)		69.6	68.5	77.5	79.7	65.3
Difficulty (P) of the items (%)	Median	75.0	72.9	82.1	86.2	67.7
	IQR	47.0	42.8	26.9	24.1	29.6
	Number of items in optimal range *p* = 40–94%	41	60	56	35	51
Discrimination index (r) of the items	Median	0.15	0.2	0.25	0.27	0.3
	IQR	0.15	0.16	0.22	0.14	0.16
	Number of items in optimal range *r* ≥ 0.2	26	46	48	38	47
Item response time (in seconds)	Median	56	56	47	57	56
	IQR	44	37	29	37	28

Overall exam difficulty is calculated as the mean value of all scores achieved by the students and is given as a percentage of the maximum score. IQR, interquartile range.

The item formats utilized in this study comprised of multiple-choice question (MCQ) in single-choice format, Kprim, Key Feature, picture diagnosis, and picture mapping, which are described in more detail in Table 2.

**Table 2:** Characteristics and scoring scheme of the five item formats used at the University of Veterinary Medicine Hannover.

Item format	Description	Evaluation
MCQ	A one-best-answer format featuring one correct option (attractor) and two to four incorrect choices (distractors).	Attractor chosen: One point. Distractor chosen: No points.
Kprim	A true-false selection item with exactly four answer options. Each of these options must be marked as “correct” or “incorrect.”	Four correct matches receive one point, three correct matches earn half a point, and less than three correct matches result in no points.
Key feature	Three individual items with a predetermined order, that are designed to build on each other regarding content, case study or topic. After response selection and choice confirmation, selected options cannot be changed.	Each correctly answered subquestion awards one point.
Picture diagnosis	A marker must be placed on a picture.	If the marker was positioned within the predefined area, one point is awarded.
Picture mapping	Terms are matched to predefined, specific marks on an image.	One point is rewarded for a completely correct assignment of the terms. Half a point is awarded if at least half of the terms were assigned correctly.

### 2.2 Log data analysis

Based on the log data, the response time per item of each examinee was determined, and subsequently, the mean response time for each individual item was calculated. For the respective subjects and examinees, the median time spent on each item and the submission time of the exam was recorded. To standardize and facilitate comparability, the submission time was calculated as a percentage of the maximum available time for the examinations.

The overall difficulty of the examinations was calculated as the mean value of all examinees’ scores and is presented as a percentage of the maximum attainable score.

For each of the 346 individual items, the parameters considered were the item format, response time, character count as well as the two psychometric characteristics: discrimination index and difficulty. The character count was defined as the sum of all letters and numbers, including special characters, punctuation, and spaces from the item stem and the answer options. These parameters were then checked for correlation. Correlations between the response time and the parameters difficulty, discrimination index and character count were examined separately for every item format in order to exclude the influence of the variances between the formats. Only the statistical data for the two formats MCQ and Kprim are presented, as a sufficiently high number of items for a meaningful statistical evaluation of the other formats was not achieved. As a next step, the ratio between the length of the question stem and the answer options was examined. For this purpose, items of the two item formats MCQ (*n* = 231) and Kprim (*n* = 81) were considered. The relative proportion of the character count of the question stem to the total character count of the item was calculated for each, and its relationship with the parameters difficulty and discrimination index was assessed.

Regarding the response selection behavior, data on changes made to the selected answer option were only available for items of the MCQ format. This meant that a total of 231 items from the five examinations were available for evaluation. For every item, the number of changes in answer selection, along with the corresponding switch between the originally selected option and the newly chosen answer were recorded for each examinee. Furthermore, in cases where the response selection was changed several times, the last change was identified, as it was the one that was ultimately graded. Due to variations in the exam conditions concerning item and participant numbers, the analyses were conducted based on relative proportions. For each exam, the following parameters were calculated:

1. The proportion of items where the chosen answer option was changed by at least one examinee.
2. The proportion of examinees who altered the originally selected option to a different response for at least one item.
3. The average proportion of items in which individual examinees modified the original answer.

To assess the quality of answer modifications in detail, the number of changes between distractors and attractor or between different distractors was examined. For clarity, distractors are referred to as incorrect answer options (incorrect) and the attractor as the correct answer option (correct).

### 2.3 Difficulty and discrimination index

The difficulty of an item is defined as the percentage of participants who answered the task correctly and can thus range from 0 to 100% (11). The recommended range for item difficulty is 40–94% (12). Discrimination index describes an item’s ability to differentiate between participants with high performance and those with low performance. Items with good discrimination are answered correctly by good candidates and incorrectly by poorer candidates (13). The values of the discrimination index can vary from −1 to +1, with values above 0.2 considered adequate (12).

### 2.4 Statistics and data privacy

This study was approved in advance by the Data Protection Officer at the TiHo. All utilized and collected data were processed and analyzed anonymously. Students had to agree a data protection declaration upon matriculation, which permits the use of data collected during examinations in anonymized form in accordance with the requirements of Art. 6(1)(e), 89 GDPR in conjunction with § 13 Lower Saxony Data Protection Law (Niedersächsisches Datenschutzgesetz, NDSG).

Access to the raw data was restricted to the authors of this paper only, and all data was stored and processed on secure servers within the institution. To protect students’ data privacy, all personal identifiers were removed from the data before analysis, including matriculation numbers and any other information that could potentially be used to identify individual students. Instead, a unique, anonymized identifier was assigned to each data point to maintain the integrity of the dataset. Descriptive and statistical analysis was performed using aggregated data to prevent the identification of students based on their response behavior during the exams.

The descriptive analysis was performed using the spreadsheet software Microsoft® Office Excel 2010 (Microsoft Corporation, Redmond, WA, USA), while advanced statistical analysis was conducted using SAS® Software, Version 9.4, and SAS® Enterprise Guide® 7.1 (SAS Institute Inc., Cary, NC, USA).

Concerning quantitative data, all normally distributed numerical values are presented as mean values, including the standard deviation (SD) where applicable. For non-normally distributed values, the median and interquartile range (IQR) are provided.

To examine the correlations among the quantitative parameters, these were initially tested for normal distribution using the Kolmogorov–Smirnov test. Subsequently, a Spearman’s rank correlation analysis was performed on non-normally distributed data. For correlations between qualitative with quantitative parameters, the Kruskal-Wallis test was applied, followed by the Dwass-Steel-Critchlow-Fligner pairwise comparison method. A significance level of 5% was used, indicating that a *p*-value <0.05 implied that the influence of the parameters was significant.

## 3 Results

### 3.1 Time of submission of the exams

**Figure 1:**
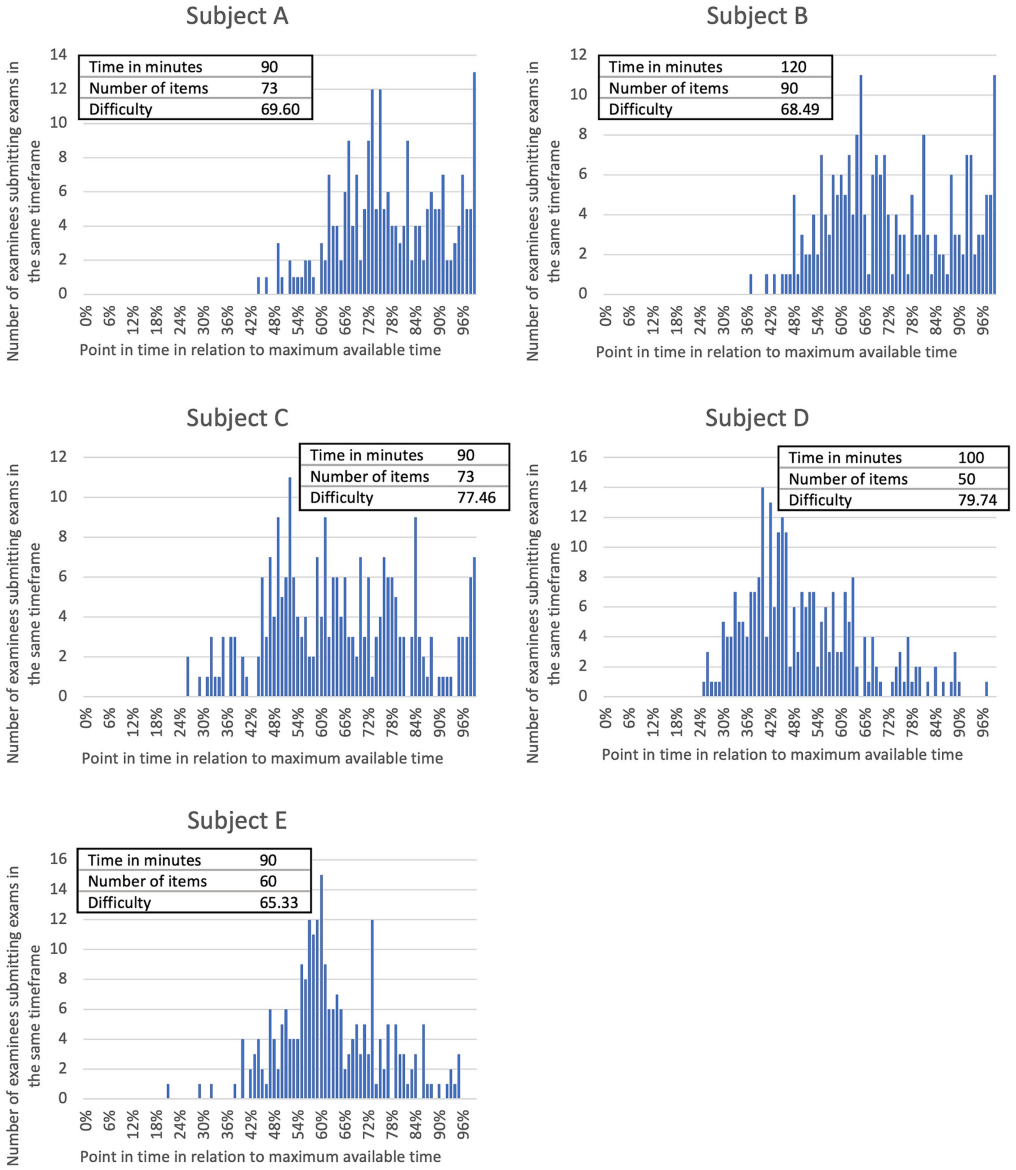
Absolute number of examinees submitting their exams at the same point in time for each of five anonymized academic subjects of veterinary medicine **(A–E)** including information on number of items and exam time limit. Submission times of exams by individual students were calculated as a percentage of the maximum available exam time. Examination difficulty is determined as the mean examination score of all examinees of the respective exam and shown as a percentage of the highest achievable score. Spearman’s rank correlation analysis shows a significant (*p* < 0.0001) negative relationship (*r*_s_ = −0.2738) between examination difficulty and submission time of the students.

Log data was initially analyzed by examining the time of submission of exams by the participants in their respective subjects. The absolute number of submissions per submission time, along with the available time in minutes, the number of items, and the overall difficulty of each exam are presented in Figure 1. Across all five of the analyzed exams, half of the candidates completed the exams within 64% (IQR: 11%, range: 46–77%) of the maximum available time. Three out of four students had submitted their exams within 77% (IRQ: 17%, range: 58–90%) of the exam time. Additionally, 90% of the participants finished the exams within 92% (IQR: 17%, range: 70–98%) of the exam time.

For each exam, the average allocated time per item was calculated for further analysis. Subsequently, correlations among the parameters “available time per item,” “submission time,” and “overall difficulty” were examined. A significant (*p* < 0.0001) negative correlation (*r*_s_ = −0.4312) was observed between the available time per item and the submission time. Hence, participants did not utilize the additional available time per item in exams that allocated more time per item.

Furthermore, a significant (*p* < 0.0001) positive correlation (*r*_s_ = 0.1106) was found between the available time per item and overall exam difficulty. This implies that the more time students had to answer the items, the higher the frequency of correct answers. There existed a significant (*p* < 0.0001) negative correlation (*r*_s_ = −0.2738) between the submission time and overall exam difficulty. Accordingly, students completed easier exams earlier.

### 3.2 Item response time

The median item response time including range for each of the five academic subjects is shown in Table 1.

**Figure 2:**
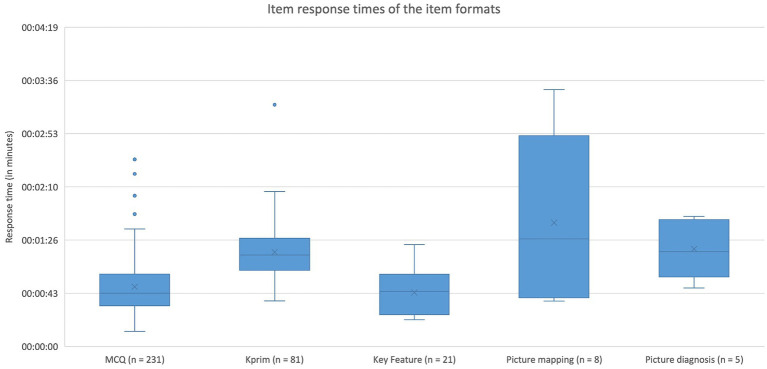
Item response times of the five item formats MCQ, Kprim, Key Feature, picture diagnosis, and picture mapping. Kruskal-Wallis test displays significant differences (*p* < 0.0001) between the response times of item formats. Pairwise comparisons using the Dwass-Steel-Critchlow-Fligner method reveal statistically significant differences between MCQ and Kprim (*p* < 0.0001) as well as between Key Feature and Kprim (*p* < 0.0001); *n* = 346.

Regarding the five item formats MCQ, Kprim, Key Feature, picture mapping, and picture diagnosis, the median response time was evaluated. For items of the MCQ format (*n* = 231), 43 s (IQR: 26 s) were spent, for Kprim (*n* = 81) 74 s (IQR: 26 s), for Key Feature per subquestion (*n* = 21) 44 s (IQR: 30 s), for picture mapping (*n* = 8) 87 s (IQR: 84 s), and for picture diagnosis (*n* = 5) 77 s (IQR: 35 s). The effect of item format on item response time was significant (*p* < 0.0001). A more detailed distribution of the required time per item and format is depicted in Figure 2. Pairwise comparisons show significant differences between the formats MCQ and Kprim (*p* < 0.0001) as well as between Key Feature and Kprim (*p* < 0.0001). There are no significant differences between any of the other formats. Further analysis reveals significant differences between item formats referring to their difficulty (*p* = 0.0003) and discrimination index (*p* = 0.0243). Pairwise comparisons show significant variations in difficulty for MCQ and Kprim (*p* = 0.0009) as well as for Kprim and Key Feature (*p* = 0.0026). For variations in discrimination index the test was significant for MCQ and Key Feature (*p* = 0.0072) as well as Kprim and Key Feature (*p* = 0.0228).

**Figure 3:**
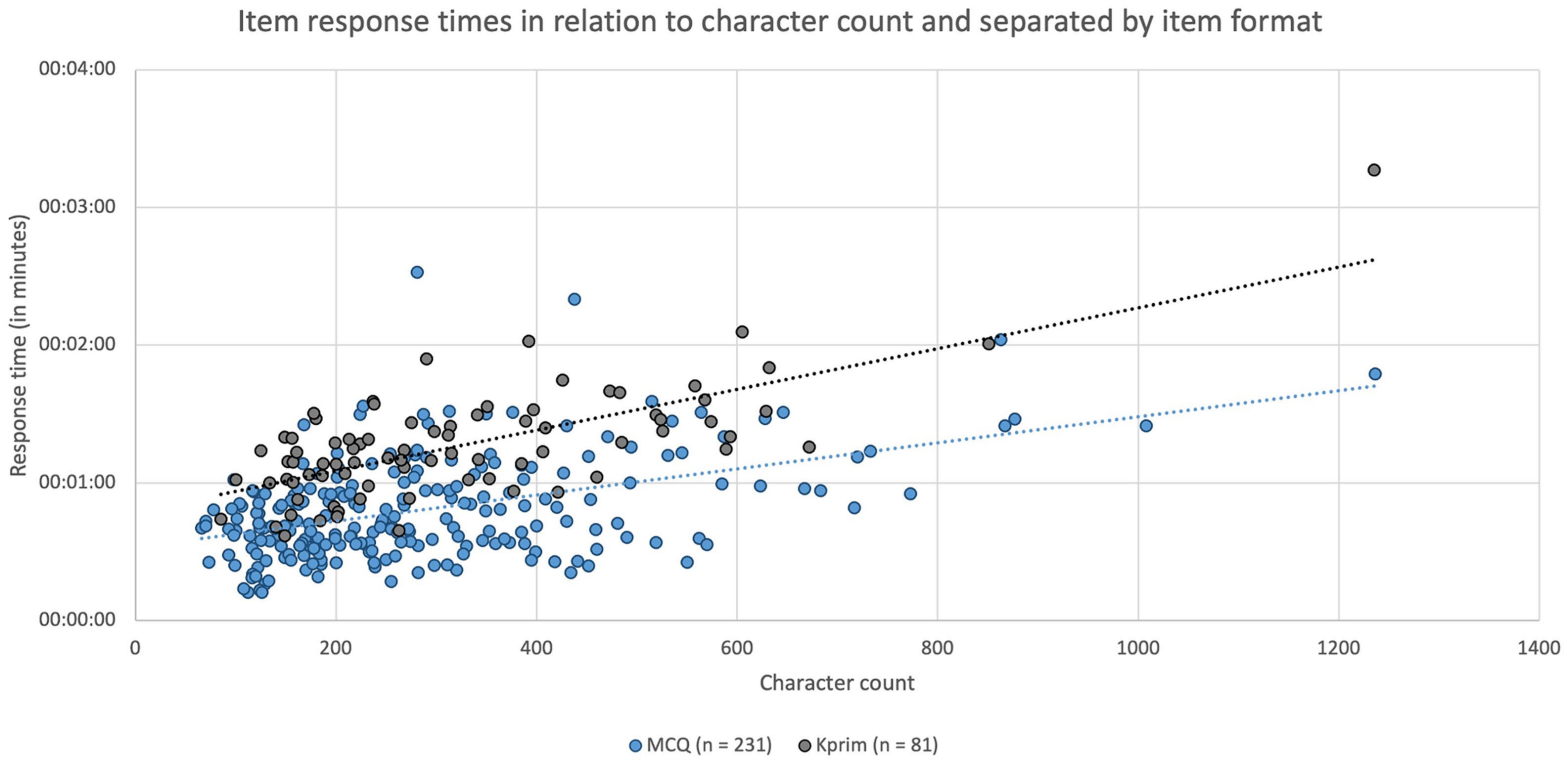
Item response times in minutes depending on the respective character count and separated by item format MCQ (*n* = 231, blue) and Kprim (*n* = 81, gray). Spearman’s rank correlation analysis indicates a statistically significant positive correlation between item response time and character count for MCQ (*p* < 0.0001, *r*_s_ = 0.3809) and Kprim (*p* < 0.0001, *r*_s_ = 0.5986); *n* = 312.

In addition to the influence of the item format on item response time an assessment of the impact of the character count of an item was included. Median character count of the item formats was 246 characters (IQR: 200 characters) for MCQ items (*n* = 231), 268 characters (IQR: 222 characters) for Kprim (*n* = 81), 446 characters (IQR: 243 characters) per subquestion for Key Feature (*n* = 21), 253 characters (IQR: 113 characters) for picture mapping (*n* = 8), and 292 characters (IQR: 109 characters) for picture diagnosis (*n* = 5). Variations in character count of the various item formats were significantly different (*p* = 0.0003) but only for the pairwise comparison of MCQ and Key Feature (*p* < 0.0001). In Figure 3 the formats MCQ (*n* = 231) and Kprim (*n* = 81) were included separately to display the time spent on each item based on individual character count. A significant positive correlation was found between the two parameters item response time and character count for both MCQ (*p* < 0.0001, *r*_s_ = 0.3809) and Kprim (*p* < 0.0001, *r*_s_ = 0.5986), which indicated that more time is needed to complete items the more characters they contain.

**Figure 4:**
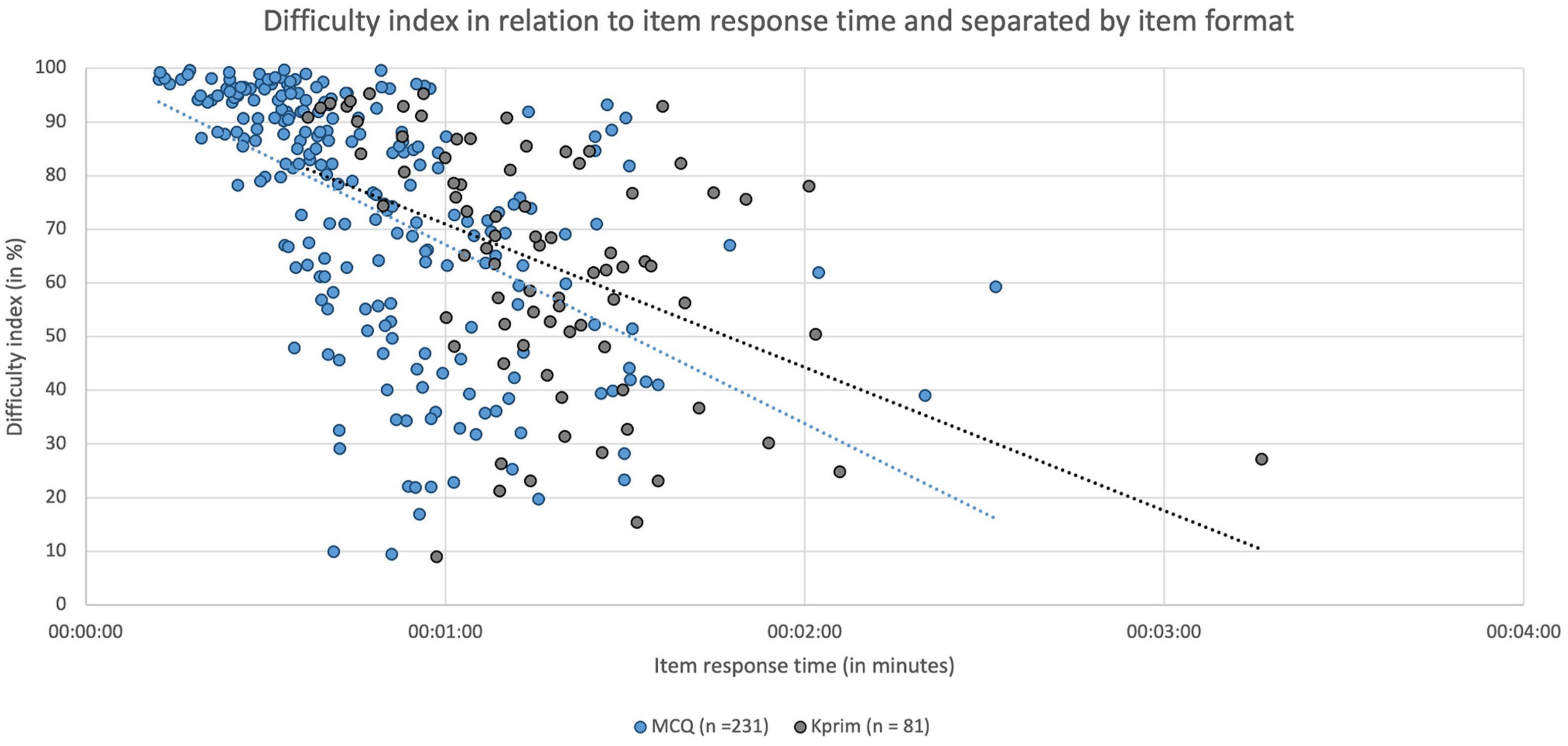
Item response times in minutes depending on the respective item difficulty index and separated by item format MCQ (*n* = 231, blue) and Kprim (*n* = 81, gray). Spearman’s rank correlation analysis indicates a statistically significant negative correlation between item response time and difficulty index for MCQ (*p* < 0.0001, *r*_s_ = −0.6607) and Kprim (*p* < 0.0001, *r*_s_ = −0.5038); *n* = 312.

Figure 4 illustrates both the difficulty of individual items separated by item format and their respective average response time, revealing a significant and negative relationship between the parameters “difficulty” and “item response time” for MCQ (*p* < 0.0001, *r*_s_ = −0.6607) and Kprim (*p* < 0.0001, *r*_s_ = −0.5038). Accordingly, answering more difficult questions required more time.

However, no significant correlation (MCQ: *p* = 0.2101; Kprim: *p* = 0.7390) was found for the relationship between character count and difficulty.

**Figure 5:**
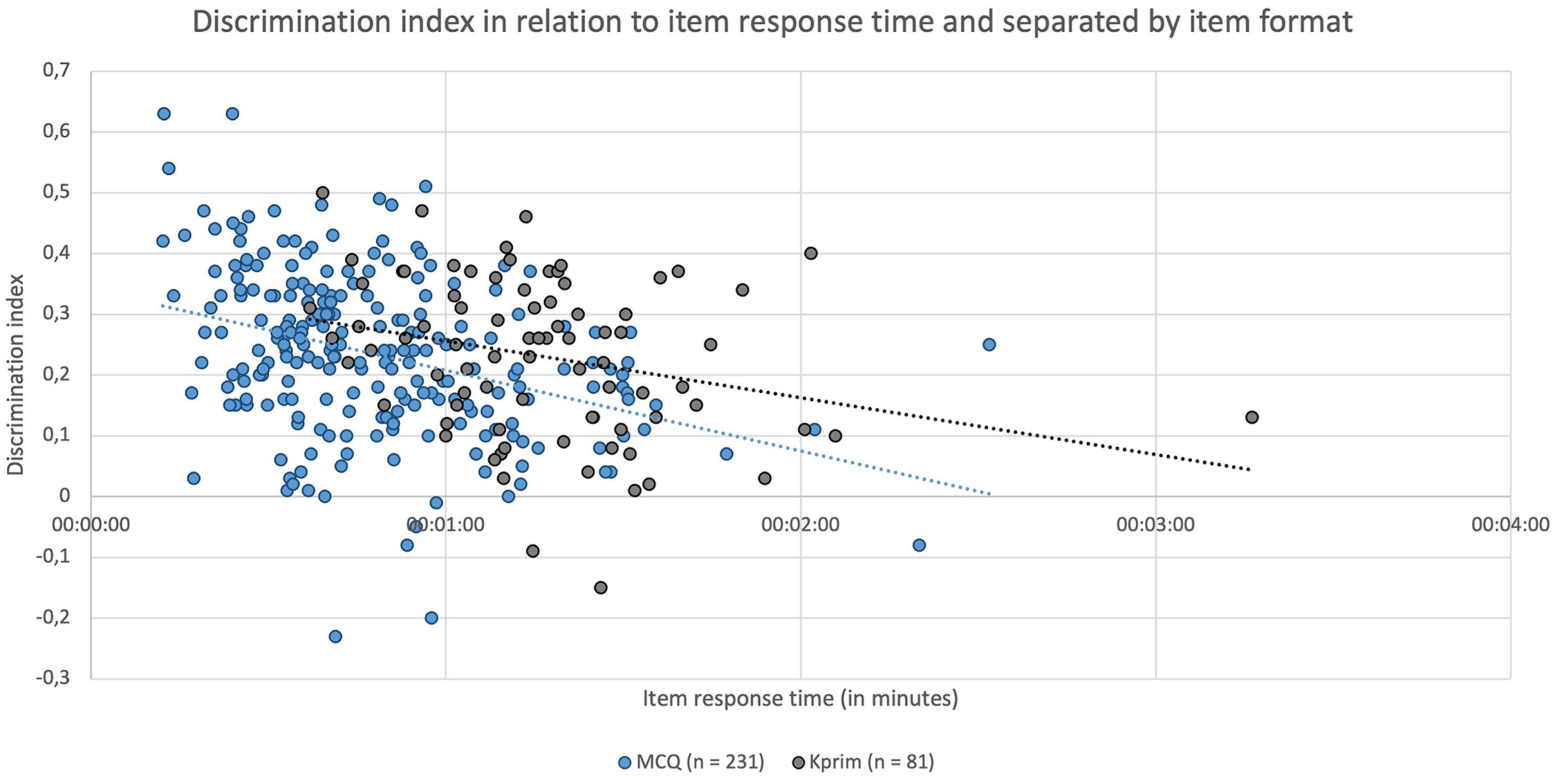
Item response times in minutes depending on the respective item discrimination index and separated by item format MCQ (*n* = 231, blue) and Kprim (*n* = 81, gray). Spearman’s rank correlation analysis shows a statistically significant negative correlation between item response time and discrimination index for MCQ (*p* < 0.0001, *r*_s_ = −0.3779) and Kprim (*p* = 0.0099, *r*_s_ = −0.2851); *n* = 312.

Figure 5 depicts the discrimination index of individual items separated by item format and their respective average response time. A significant negative correlation was identified between the parameters “discrimination index” and “item response time” for MCQ (*p* < 0.0001, *r*_s_ = −0.3779) and Kprim (*p* = 0.0099, *r*_s_ = −0.2851). This indicates that questions on which students spent more time generally showed poorer discrimination, suggesting that the time students took to respond to an item might be linked to its ability to discriminate effectively between high- and lower-performing students.

**Figure 6:**
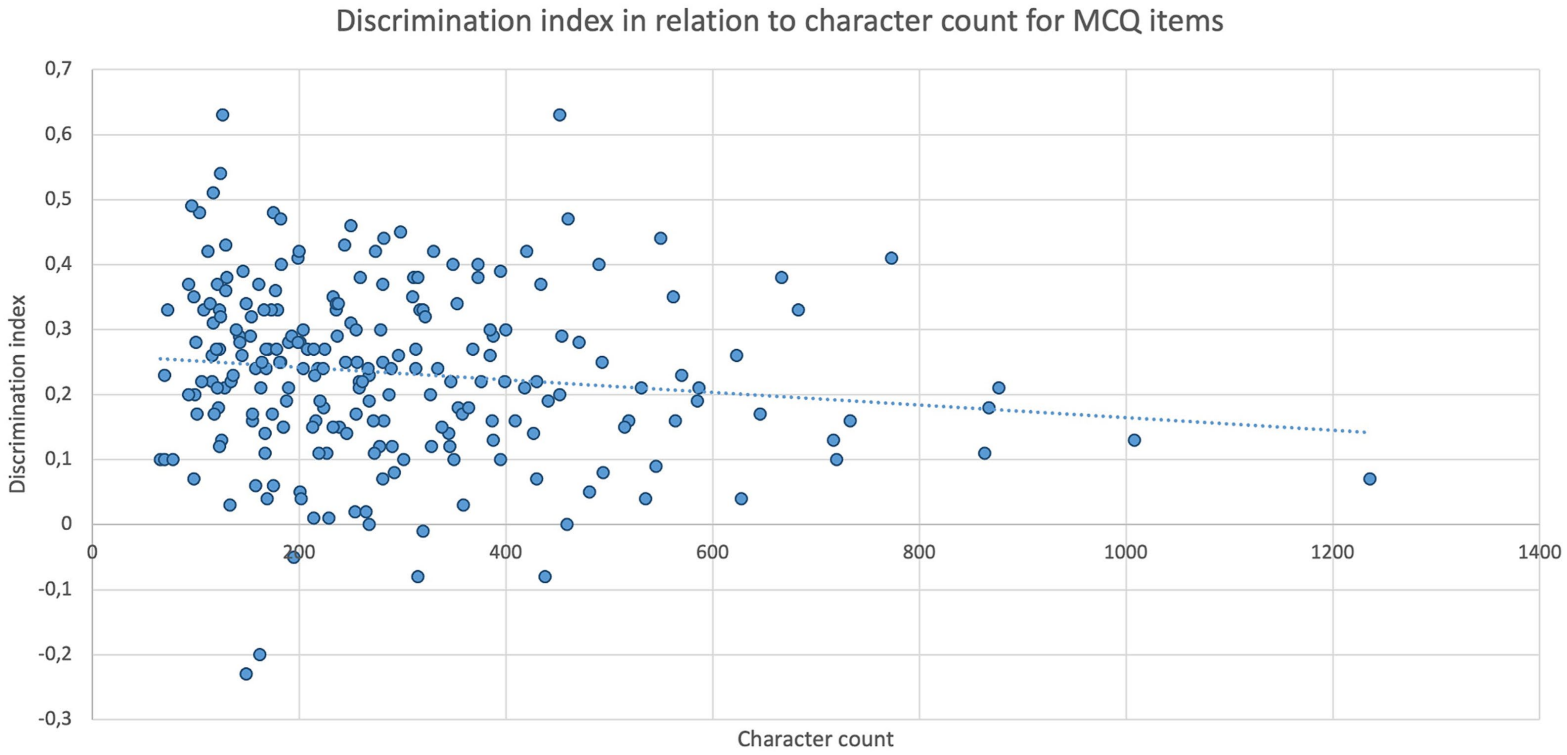
Item discrimination indexes of MCQ items (*n* = 231) depending on their respective character count. Spearman’s rank correlation analysis displays a statistically significant negative correlation between discrimination index and character count (*p* = 0.0236, *r*_s_ = −0.1496); *n* = 231.

Furthermore, Figure 6 depicts the discrimination index of individual MCQ items in relation to their respective character count, where a significant (*p* = 0.0236) negative correlation (*r*_s_ = −0.1496) was found for both parameters. Consequently, the discrimination index tended to decrease slightly for MCQ items with a higher character count. However, for Kprim items, no correlation (*p* = 0.5534) between the discrimination index and character count could be verified.

Regarding the evaluation of the effect of the length of the question stem, no significant correlation was found between the parameters “relative proportion of the question stem” and “difficulty” for MCQ (*n* = 231, *p* = 0.5627) and Kprim (*n* = 81, *p* = 0.619) nor for “relative proportion of the questions stem” and “discrimination index” for both MCQ (*n* = 231, *p* = 0.9718) and Kprim (*n* = 81, *p* = 0.4449).

### 3.3 Response selection behavior

Table 3 shows the calculated parameters concerning the relative proportions of changes made to the chosen answer option for each of the five subjects.

**Table 3:** Evaluation of the response selection behavior of multiple choice questions (*n* = 231) separated by examination subject (A–E), including an overall average of all exams.

	Subject A (*n* = 50)	Subject B (*n* = 68)	Subject C (*n* = 46)	Subject D (*n* = 25)	Subject E (*n* = 42)	Average (*n* = 231)
Proportion of items where response selections were changed	89.6%	98.4%	90.2%	92.0%	100%	94.04%
Percentage of examinees who changed at least one of their originally selected answers to a different option	88.6%	92.7%	79.8%	67.1%	96.2%	84.88%
Average proportion of items of the exam for which an examinee changed their response	6.25%	6.45%	4.88%	4.00%	10.0%	6.32%

The relative proportions of changes between incorrect and correct options are presented for each individual exam in Table 4. The mean values for the changes were 42.89% (SD ± 3.81%) for incorrect to correct ones, 27.34% (SD ± 6.71%) for incorrect to incorrect ones, and 29.77% (SD ± 4.01%) for correct to incorrect ones.

**Table 4:** Evaluation of the quality of response selection modifications separated by corresponding examination subject.

Subject	Incorrect to correct (in %)	Incorrect to incorrect (in %)	Correct to incorrect (in %)
A	40.42	28.74	30.84
B	43.55	25.05	31.40
C	43.64	21.59	34.77
D	49.12	21.64	29.24
E	37.73	39.67	22.60

## 4 Discussion

### 4.1 Time of submission of the exams

Within this study, log data of electronic examinations at the TiHo as well as the response selection behavior of the examination participants were evaluated for the first time in order to examine whether recommendations for the design of online examinations can be derived from them.

When examining the submission times of participants for different exams, a wide range of exam completion times becomes evident. Within a time frame of 58–90% of the maximum time limit, 75% of the participants managed to complete their exams. The time range for 90% of the students was between 70 and 98% of the available time. This highlights that the time required for exam completion cannot be reliably standardized. However, trends can be derived from the statistical analyses conducted. Notable was the observation that participants tended to finish exams earlier in relation to the maximum available time when there was more time allocated to answer individual items and when the overall exam was easier.

In exams with the longest allocated time to complete items in subjects D (120 s per item) and E (90 s per item), it is apparent that students generally did not utilize this longer time frame. The majority of candidates managed to complete the exam before 70% of the exam time had elapsed. Thus, theoretically, there is room to reduce the time allocated per item from the 90 s per item recommended in the literature (12, 14) and used by the TiHo. However, this reduction needs to consider the correlation between the available exam time and the overall difficulty of the exams reported in this study, as students tend to answer items more accurately when they are granted more time per item, which is also described in Lovett’s publication (15). The positive impact of additional time per item on students’ performance was already demonstrated in previous studies (16–19). Here, the effects are most apparent for students with relatively weaker performance (16, 17, 19), or those experiencing test anxiety (20). This effect might be attributed to reduced exam stress (21), more time to consider items, ample time for item completion (22) as well as reduced test anxiety (20, 21), which is said to lead to difficulties in concentration and impaired information processing skills (20). In this context, a reduction in exam time should be carefully considered with regard to exam fairness. Particularly with regard to the mentioned exam fairness, it must be noted that increasing time pressure can put certain other groups of examinees at a disadvantage. For example, studies indicate that increasing time pressure has a more substantial negative impact on the performance of female examinees than male participants (23, 24). Furthermore, students with learning, cognitive or psychiatric disabilities such as ADHD (15, 25), Asperger’s syndrome (25) or dyslexia (9) are entitled to accommodations for disadvantages, which is often in the form of an extended time limit (15), since the negative impact of time pressure is especially evident for students with disabilities (26).

### 4.2 Item response time

Examining the actual time taken by candidates to complete the exams on the level of individual items also indicates that candidates did not utilize the time limit of 90 s per item. The average response time ranged between 47 and 57 s per item for all veterinary subjects. This observation aligns with the findings of other analyses, consistently reporting a time of 60 s or less per multiple-choice item (27–30). In some cases, the required response time per item was approximately 40 s or less (28–30). Therefore, there is an opportunity of reducing the exam time limit to 60 s per item, a practice already standard in some other exams (28, 31, 32). On the one hand, this would allow more items to be included in the same time frame, thus improving the validity and reliability of exams (12, 33–37), and, on the other hand, a stricter time limit can be used as a tool to reduce interchange between candidates in unproctored online examinations (9, 38).

It is important to note that three additional aspects need to be considered when determining time limits for exams and individual items.

Firstly, exams in the medical field are intended to test students’ knowledge, understanding, and application of knowledge and should thus be conducted as power tests (12). When time constraints are introduced or exams are conducted under high time pressure, factors such as candidates’ stress resistance and cognitive performance affect overall performance (9, 12). Consequently, the difficulty of exams increases (39, 40), and a potential time shortage can result in unanswered questions or blind guessing, raising concerns about the validity of exam results (12, 22, 41). In light of the examination objective in veterinary medicine, so-called speed tests where the time factor significantly impacts candidates’ performance should be avoided.

Secondly, the influence of exam time limits on performance, primarily observed in weaker students, should be considered to preserve exam fairness. A shorter time frame can lead to poorer test results.

Lastly, the ratio of different item formats should be mentioned (42) since significant differences in response time for formats were shown. Especially for answering the three formats Kprim (74 s), picture diagnosis (77 s), and picture mapping (87 s), students needed on average more than 60 s of response time, while MCQ (43 s) and Key Feature (44 s per subquestion) required less than 60 s. Other studies also conclude that formats like picture diagnosis and picture mapping are more time-consuming for candidates than MCQ (43, 44). Therefore, when determining the time limits for exams, it is essential to consider the item formats used and their ratio (42). This ratio is particularly important if aiming for the aforementioned reduction of response time to 60 s per item. Since students require significantly longer than 60 s on average for Kprim and image-based formats, there is a risk that an exam with a high proportion of these formats becomes a speed test. Hence, when limiting it to 60 s per item, attention should be given to predominantly select MCQ items, as less than 60 s are sufficient for this format, thereby balancing the additional time required for Kprim and image-based formats.

Furthermore, such exams can be evaluated to determine whether time significantly impacts students’ performance. The Education Testing Service defines a multiple-choice exam as a power test if all participants answer at least 75% of all items, and 80% of participants respond to all of the items (45). Otherwise, it is considered a speed test if these criteria are not met. However, this rule of thumb was developed based on paper-based exams and the assumption that examinees do not complete the exam if they run out of time. In the context of electronic multiple-choice tests where an incorrect answer does not result in a negative grading, it is more likely that examinees randomly select answer options for the remaining items to still have a chance to randomly choose the correct option. This is referred to as rapid guessing behavior (22, 44). Modern analysis methods use the log data from electronic assessments to identify rapid guessing behavior. Schnipke (22) graphed the standardized natural logarithm of response time to detect examinees with accelerated response times and a lower frequency of correct answers, indicating that these students might be running out of time. In addition, other complex models and methods, including those based on Item Response Theory, have been developed to assess the time influence on students’ exam performance and behavior (41, 44).

Regarding the analysis of character counts, response time, and item parameters of the tasks and their correlations, it was demonstrated that students need more time for items with high difficulty, poor discrimination index, and a higher number of characters. Additionally, it was shown that MCQ items with a higher character count tend to have a poorer discrimination index, but that the character count has no effect on difficulty. These effects have been described in other studies that also concluded that examinees require more time for poorly discriminating items (30, 46) as well as for difficult items (46, 47). As a result, to improve discrimination and reduce required response time, items should be kept as clear and concise as possible, which aligns with the formal requirements of multiple-choice items in the literature (12, 28, 48–52). However, focusing on the brevity of items should not be at the expense of the learning content to be assessed, as it is outlined in Bloom’s taxonomy (53).

Bloom’s taxonomy categorizes cognitive skills into six levels: remembering, understanding, applying, analyzing, evaluating, and creating (53). Questions designed to test basic recall or understanding can indeed be kept short and precise, reducing both the time needed for students to respond to these items and the time required for question authors to create them. For example, a straightforward multiple-choice question requiring students to recall a specific fact or definition can be brief without sacrificing its effectiveness. In contrast, questions that aim to assess higher-order cognitive skills, such as applying knowledge to new situations, analyzing data, or evaluating concepts, often necessitate a more detailed question stem (50, 52). For instance, regarding the clinical sciences of veterinary medicine, presenting a comprehensive scenario that provides sufficient context to assess students’ critical thinking and problem-solving abilities is essential. Such scenarios might include clinical findings, laboratory results or detailed case vignettes (50). These elements are crucial for testing students’ abilities to integrate and apply their knowledge but inevitably lead to an increased character count of these items. Subsequently, a careful balance is necessary when creating questions that are able to assess these higher-level cognitive skills. While the objective is still to keep items as concise as possible, it is equally important to ensure that all necessary information is included to allow students to fully understand and respond to the question.

Consequently, an efficient question design is recommended to reduce the time needed for authors to create items and for students to answer them, which can make it feasible to reduce the time per item and thus include more items in the same timeframe as before, which positively impacts the quality criteria of exams (52).

Applying these findings to the design of online assessments, a time limit of 60 s can be set for this format as long as the exam is primarily composed of MCQs and Key Feature items, which are short and concise. The shorter allocated time per item can help reduce the risk of cheating. However, it is essential to ensure that students do not feel overly rushed. Some authors even discuss explicitly designing exams to create significant time pressure on participants as a means to reduce cheating attempts (9, 38, 54). This statement should be viewed critically based on the aspects discussed earlier. When it comes to students with disabilities (see section 4.1), reducing the time per item must be approached with caution, as this can create additional challenges in administering exams (15, 25). In compliance with local laws and regulations, appropriate accommodations must be provided, which typically involve extending the exam time to allow students to utilize these accommodations effectively (15). Decision-making processes should be established to determine whether, and to what extent, accommodations are offered to disadvantaged students, as well as how much extra time is actually necessary (15). Additionally, the technical and organizational challenges of granting extra time to only some students must be considered (25).

### 4.3 Response selection behavior

The analysis of the log data from the five exams indicates that for almost all of the items there was at least one student changing their answer and that the majority of students belonged to the group that revisited and modified the originally selected response option. However, it is noteworthy that each individual student changed their originally selected answers only for a very small proportion of items. These findings based on the data from veterinary students corroborate the results of other studies, which also conclude that nearly all students changed their original answers to a different one, yet each individual participant only makes corrections for a small fraction of all exam items (55).

One potential influencing factor on this low frequency of modified answers could be the widespread belief that the initial intuition when reading the question is the correct answer. Benjamin et al. (55) reported in their literature review that almost all students believe that revising their answer does not impact the exam result; 75% were even of the opinion that the result would deteriorate. The teaching staff shared a similar belief, with over half of the respondents stating that exam results would worsen due to answer modifications (55). In contrast, studies demonstrate that the majority of answer revisions are from incorrect to correct responses, resulting in improved exam results (55–60). Hence, this phenomenon is termed the “first instinct fallacy” (55, 57–59). The analysis of the response selection behavior in this study reaches the same conclusion, as the majority of all corrections were either from incorrect to correct options or between two different incorrect options, resulting in a positive or neutral impact on exam scores.

Accordingly, the available time for exams should not be stipulated within such a short timeframe that participants lose the opportunity to adjust their response selections due to time constraints. For online examinations, one study recommends that students should only be allowed to select an answer for an item once only (38). We do not follow this recommendation based on the results of our analysis, as not being able to change the originally selected answer could potentially worsen the exam results.

As an alternative, a so-called “block setting” can be used for electronic exams. In this approach, the exam is divided into equally sized groups of items, and the items within the blocks as well as the blocks themselves can be individually randomized for each examinee. Access is only granted to the items in the currently worked-on block, which can be accessed and modified multiple times. After completing the block, the following block is unlocked and the previous item block is locked. This means that students no longer have access to the previously worked-on items. This approach allows exams to be structured better, the students focusing their concentration on the items within the block. Randomizing the blocks and the included items can be used to reduce cheating (38).

### 4.4 Implications and limitations of the study

A limitation of this study was the small sample size for the image-based item formats, namely picture diagnosis and picture mapping. The reason for that was that for the sake of comparability exams were chosen in which all five item formats were used and had already been established for several years. However, the image-based formats were less frequently employed in these exams. In future studies, a greater emphasis should be placed on a more in-depth evaluation of the two formats picture diagnosis and picture mapping.

Since the focus was primarily on analyzing log data and response selection behavior of electronic examinations, more research is needed regarding the evaluation of the psychometric quality of item formats in online examinations and thereby identify suitable formats for distance exams. As both lecturers and students in veterinary medicine call for more application- and competence-based item formats (61, 62), these would be of particular interest, for example key feature and the image-based formats referenced in this work.

Regarding the items included in this study and their item analysis parameters, it is noticeable that some items display a low discrimination index, and a few items exhibit a negative discrimination index (see Figure 5). This indicates that these items might not effectively differentiate between higher-performing and lower-performing students, according to Krebs (12) as well as McCowan and McCowan (13). Negative discrimination indices imply that students understood these items in the opposite way than originally intended, meaning good students answered incorrectly while lower-performing students chose the correct answer (12). Therefore, such a negative discrimination index is an indicator of potential flaws in item construction or a lack of alignment with the learning objectives. To address this issue, the content of items with negative discrimination was reviewed for correctness, relevance, formal errors, and cueing, including a distractor analysis. However, no significant issues were identified. The low discrimination indices can partly be attributed to some items being relatively difficult (*p* < 40%) and some items being relatively easy (*p* > 80%) (see Figure 4). The best discrimination indices are found in items with medium difficulty, and deviations upwards or downwards result in significantly poorer discrimination values (12). Regardless of the reason, it must be noted that such items directly impact the calculated correlations of this study.

Open-book exams are a format commonly used for electronic distance assessments. Over the course of the COVID-19 pandemic, they gained importance as online proctoring is not necessarily required for this format (4), which resolves data protection issues associated with remote examinations. Comparative analyses already showed that the psychometric parameters of closed-book in-person exams and online open-book exams do not significantly differ (54, 63–65). Hence, according to the mentioned literature, this innovative format can be considered suitable for summative assessments. However, it is crucial to take into account the current developments in the field of artificial intelligence (AI). Generative AIs, particularly ChatGPT, are capable of successfully passing challenging final and licensure exams, including several law bar exams (66), the United States Medical Licensing Exam (67), the German medical state exam (68), and other assessments (69, 70). In Progress Tests, ChatGPT answered over 60% of the items correctly (71). It can be assumed that this performance can also be reproduced in the field of veterinary medicine. Given that such AIs are readily available to students as well (72), the feasibility of distance assessments needs to be critically examined and re-evaluated (72–74).

## Data availability statement

The datasets presented in this article are not readily available because they include legally protected personal and examination information. Requests to access the datasets should be directed to RR, richter.robin@tiho-hannover.de.

## Ethics statement

This study was conducted according to the ethical standards of the University of Veterinary Medicine Hannover, Foundation. The doctoral thesis committee of the university, which acts as the university’s ethics committee, validated the project in accordance with ethical guidelines regarding research with human participants and approved the study.

## Author contributions

RR: Conceptualization, Investigation, Methodology, Writing – original draft, Writing – review & editing. AT: Writing – review & editing, Funding acquisition, Supervision. ES: Supervision, Writing – review & editing, Funding acquisition, Methodology, Project administration.

## References

[1] Marinoni G Vant Land H Jensen T. The impact of Covid-19 on higher education around the world. IAU Global Survey Rep. (2020) 23:1–17.

[2] Seyfeli F Elsner L Wannemacher K. Vom Corona-Shutdown Zur Blended University?: Expertinnenbefragung Digitales Sommersemester. 1st ed. Baden-Baden, Germany: Tectum (2020).

[3] Wissing F. Digitale Lehre für alle: Voraussetzungen, Machbarkeit und Optionen im Human- und Zahnmedizinstudium. Medizinischer Fakultätentag. (2020). Available at: https://medizinische-fakultaeten.de/wp-content/uploads/2020/10/MFT-und-GMA-Positionspapier-zu-digitalen-Lehr-und-Pru%CC%88fungsformaten.pdf

[4] Routh J Paramasivam SJ Cockcroft P Nadarajah VD Jeevaratnam K. Veterinary education during Covid-19 and beyond-challenges and mitigating approaches. Animals. (2021) 11:1818. doi: 10.3390/ani11061818, PMID: 34207202 PMC8234198

[5] Gnewuch L. Digitalisierung der Lehre– Situationsanalyse und Perspektiven in der Veterinärmedizin. [Dissertation]. Berlin, Germany: Freie Universität Berlin (2023).

[6] Naundorf H. Untersuchung der Hybridsemester-Lehre während der Covid-19 Pandemie an der Stiftung Tierärztliche Hochschule Hannover. [Dissertation]. Hannover, Germany: Stiftung Tierärztliche Hochschule Hannover (2023).

[7] Gatti T Helm F Huskobla G Maciejowska D McGeever B Pincemin J-M. Practices at Coimbra group universities in response to the COVID-19: a collective reflection on the present and future of higher education in Europe. (2020). Available at: https://www.coimbra-group.eu/wp-content/uploads/Final-Report-Practices-at-CG-Universities-in-response-to-the-COVID-19-3.pdf

[8] Thiel B. Eckpunkte Für Datenschutzkonforme Online-Prüfungen an Niedersächsischen Hochschulen. Hannover. (2021). Available at: https://lfd.niedersachsen.de/startseite/themen/weitere_themen_von_a_z/hochschulen/eckpunkte_fur_die_datenschutzkonforme_durchfuhrung_von_online_prufungen/

[9] Stadler M Kolb N Sailer M. The right amount of pressure: implementing time pressure in online exams. Distance Educ. (2021) 42:219–30. doi: 10.1080/01587919.2021.1911629

[10] Ehlers JP Carl T Windt K-H Möbs D Rehage J Tipold A. Blended Assessment: Mündliche Und Elektronische Prüfungen Im Klinischen Kontext. Zeitschrift für Hochschulentwicklung. (2010) 4:24–36. doi: 10.3217/zfhe-4-03/02

[11] Thorndike RM Cunningham GK Thorndike RL Hagen EP. Measurement and evaluation in psychology and education. 5th ed. New York, NY, England: Macmillan Publishing Co, Inc (1991). 544 p.

[12] Krebs R. Prüfen Mit Multiple Choice. Kompetent Planen, Entwickeln, Durchführen Und Auswerten. 1st ed. Bern, Austria: Hogrefe (2019).

[13] McCowan RJ McCowan SC. Item analysis for criterion-referenced tests. New York: Center for Development of Human Services (CDHS) (1999).

[14] Harden RM. Constructing multiple choice questions of the multiple true/false type. Med Educ. (1979) 13:305–12. doi: 10.1111/j.1365-2923.1979.tb01517.x, PMID: 470653

[15] Lovett BJ. Extended time testing accommodations for students with disabilities: impact on score meaning and construct representation In: Margolis MJ Feinberg RA, editors. Integrating timing considerations to improve testing practices. Oxfordshire: Routledge (2020). 47–58.

[16] Mitchell G Ford DM Prinz W. Optimising marks obtained in multiple choice question examinations. Med Teach. (1986) 8:49–53. doi: 10.3109/01421598609036845, PMID: 3724402

[17] Bridgeman B Cline F Hessinger J. Effect of extra time on verbal and quantitative Gre scores. Appl Meas Educ. (2004) 17:25–37. doi: 10.1207/s15324818ame1701_2

[18] Cuddy MM Swanson DB Dillon GF Holtman MC Clauser BE. A multilevel analysis of the relationships between selected examinee characteristics and United States medical licensing examination step 2 clinical knowledge performance: revisiting old findings and asking new questions. Acad Med. (2006) 81:103–7. doi: 10.1097/00001888-200610001-00026 17001117

[19] Harik P Clauser BE Grabovsky I Baldwin P Margolis MJ Bucak D. A comparison of experimental and observational approaches to assessing the effects of time constraints in a medical licensing examination. J Educ Meas. (2018) 55:308–27. doi: 10.1111/jedm.12177

[20] Onwuegbuzie AJ Seaman MA. The effect of time constraints and statistics test anxiety on test performance in a statistics course. J Exp Educ. (1995) 63:115–24. doi: 10.1080/00220973.1995.9943816

[21] Portolese L Krause J Bonner J. Timed online tests: do students perform better with more time? Am J Dist Educ. (2016) 30:264–71. doi: 10.1080/08923647.2016.1234301

[22] Schnipke DL. In Paper presented at the Annual Meeting of the National Council on Measurement in Education. San Francisco, CA, USA. (1995). 2–322.

[23] Steinmayr R Spinath B. Why time constraints increase the gender gap in measured numerical intelligence in academically high achieving samples. Eur J Psychol Assess. (2019) 35:392–402. doi: 10.1027/1015-5759/a000400

[24] Voyer D. Time limits and gender differences on paper-and-pencil tests of mental rotation: a meta-analysis. Psychon Bull Rev. (2011) 18:267–77. doi: 10.3758/s13423-010-0042-0, PMID: 21327340

[25] Persike M Günther S Dohr J Dorok P Rampelt F. Digitale Fernprüfungen / Online-Prüfungen außerhalb der Hochschule In: Bandtel M Baume M Brinkmann E Bedenlier S Budde J Eugster B, editors. Digitale Prüfungen in der Hochschule. Whitepaper einer Community Working Group aus Deutschland, Österreich und der Schweiz. Berlin, DE: Hochschulforum Digitalisierung (2021). 81–91.

[26] Waterfield J West B. Inclusive assessment in higher education: a resource for change. Plymouth, UK: University of Plymouth (2006).

[27] Cui Z. On the cover: time spent on multiple-choice items. Educ Meas Issues Pract. (2021) 40:6–7. doi: 10.1111/emip.12420

[28] Brothen T. Time limits on tests: updating the 1-minute rule. Teach Psychol. (2012) 39:288–92. doi: 10.1177/0098628312456630, PMID: 38309959

[29] Schneid SD Armour C Park YS Yudkowsky R Bordage G. Reducing the number of options on multiple-choice questions: response time, psychometrics and standard setting. Med Educ. (2014) 48:1020–7. doi: 10.1111/medu.12525, PMID: 25200022

[30] Chae YM Park SG Park I. The relationship between classical item characteristics and item response time on computer-based testing. Korean J Med Educ. (2019) 31:1–9. doi: 10.3946/kjme.2019.113, PMID: 30852856 PMC6589631

[31] Renner C Renner M. How to create a good exam In: Perlman B McCann LI McFadden SH, editors. Lessons learned: practical advice for teaching of psychology. Washington, DC: American Psychological Society (1999). 43–7.

[32] McKeachie W. Teaching tips. 11th ed. Boston, MA: Houghton Mifflin Company (2002) ch. 8.

[33] Downing SM. Reliability: on the reproducibility of assessment data. Med Educ. (2004) 38:1006–12. doi: 10.1111/j.1365-2929.2004.01932.x, PMID: 15327684

[34] Möltner A Schellberg D Jünger J. Grundlegende quantitative analysen medizinischer prüfungen. GMS Z Med Ausbild. (2006) 23:11.

[35] Tavakol M Dennick R. Making sense of Cronbach’s alpha. Int J Med Educ. (2011) 2:53–5. doi: 10.5116/ijme.4dfb.8dfd, PMID: 28029643 PMC4205511

[36] Jünger J Just I. Recommendations of the German Society for Medical Education and the German Association of Medical Faculties regarding university-specific assessments during the study of human, dental and veterinary medicine. GMS Z Med Ausbild. (2014) 31:Doc34. doi: 10.3205/zma000926, PMID: 25228936 PMC4152998

[37] Kibble JD. Best practices in summative assessment. Adv Physiol Educ. (2017) 41:110–9. doi: 10.1152/advan.00116.2016, PMID: 28188198

[38] Cluskey C Jr Ehlen C Raiborn M. Thwarting online exam cheating without proctor supervision. J Acad Bus Ethics. (2011) 4:1–7.

[39] Perlini AH Lind DL Zumbo BD. Context effects on examinations: the effects of time, item order and item difficulty. Can Psychol. (1998) 39:299–307. doi: 10.1037/h0086821

[40] Lindner MA Mayntz SM Schult J. Studentische Bewertung und Präferenz von Hochschulprüfungen mit Aufgaben im offenen und geschlossenen Antwortformat. Zeitschrift für Pädagogische Psychol. (2018) 32:239–48. doi: 10.1024/1010-0652/a000229

[41] Cintron DW. Methods for measuring speededness: chronology, classification, and ensuing research and development. ETS Res Rep Ser. (2021) 2021:1–36. doi: 10.1002/ets2.12337

[42] Hsieh C. Time needed for undergraduate biomechanics exams. ISBS Proc Arch. (2018) 36:847–850.

[43] Association of Test Publishers (ATP) and Institute for Credentialing Excellence (ICE). (2017). Innovative item types: a white paper and portfolio. Available at: https://atpu.memberclicks.net/assets/innovative%20item%20types%20w.%20appendix%20copy.pdf

[44] Sireci SG Botha SM. Timing considerations in test development and administration In: Margolis MJ Feinberg RA, editors. Integrating timing considerations to improve testing practices. Oxfordshire: Routledge (2020). 32–46.

[45] Swineford F. The test analysis manual (ETS SR 74-06). Princeton, NJ: Educational Testing Service (1974).

[46] Lahza H Smith TG Khosravi H. Beyond item analysis: connecting student behaviour and performance using E-assessment logs. Br J Educ Technol. (2023) 54:335–54. doi: 10.1111/bjet.13270

[47] González-Espada WJ Bullock DW. Innovative applications of classroom response systems: investigating students’ item response times in relation to final course grade, gender, general point average, and high school act scores. Electron J Integr Technol Educ. (2007) 6:97–108.

[48] Paterson DG. Preparation and use of new-type examinations; a manual for teachers. Yonkers-on-Hudson, NY: World Book Company (1924). p. 42–66

[49] Cronbach LJ. Essentials of psychological testing. 4th ed. New York, NY: Harper & Row (1984) ch. 4.

[50] Case S Swanson D. Constructing written test questions for the basic and clinical sciences. Natl Board Exam. (2002):13–104.

[51] Haladyna TM Downing SM Rodriguez MC. A review of multiple-choice item-writing guidelines for classroom assessment. Appl Meas Educ. (2002) 15:309–33. doi: 10.1207/S15324818AME1503_5

[52] Haladyna TM Rodriguez MC. Developing and validating test items. New York, USA: Routledge (2013). doi: 10.4324/9780203850381

[53] Anderson LW Krathwohl DR Airasian PW Cruikshank KA Mayer RE Pintrich PR. A taxonomy for learning, teaching, and assessing: a revision of Bloom’s taxonomy of educational objectives. New York, NY: Longman (2001).

[54] Durning SJ Dong T Ratcliffe T Schuwirth L Artino AR Jr Boulet JR. Comparing open-book and closed-book examinations: a systematic review. Acad Med. (2016) 91:583–99. doi: 10.1097/ACM.0000000000000977, PMID: 26535862

[55] Benjamin LT Cavell TA Shallenberger WR. Staying with initial answers on objective tests: is it a myth? Teach Psychol. (1984) 11:133–41. doi: 10.1177/009862838401100303

[56] Fischer MR Herrmann S Kopp V. Answering multiple-choice questions in high-stakes medical examinations. Med Educ. (2005) 39:890–4. doi: 10.1111/j.1365-2929.2005.02243.x, PMID: 16150028

[57] Kruger J Wirtz D Miller DT. Counterfactual thinking and the first instinct fallacy. J Pers Soc Psychol. (2005) 88:725–35. Epub 2005/05/19. doi: 10.1037/0022-3514.88.5.725, PMID: 15898871

[58] Couchman JJ Miller NE Zmuda SJ Feather K Schwartzmeyer T. The instinct fallacy: the metacognition of answering and revising during college exams. Metacogn Learn. (2015) 11:171–85. doi: 10.1007/s11409-015-9140-8

[59] Möltner A Heid J Wagener S Jünger J. Beantwortungszeiten von Fragen bei einem online durchgeführten Progresstest: Abhängigkeit von Schwierigkeit, Studienjahr und Korrektheit der Antwort und die First Instinct Fallacy. Bern, Düsseldorf: Jahrestagung der Gesellschaft für Medizinische Ausbildung (GMA) (2016).

[60] AlMahmoud T Regmi D Elzubeir M Howarth FC Shaban S. Medical student question answering behaviour during high-stakes multiple choice examinations. Int J Technol Enhanc Learn. (2019) 11:157–71. doi: 10.1504/IJTEL.2019.098777, PMID: 21607743

[61] Ehrich F. Untersuchungen zu kompetenzorientierten Prüfungen an der Stiftung Tierärztliche Hochschule. [Dissertation]. Hannover, Germany: Tierärztliche Hochschule Hannover (2019).

[62] Schaper E Tipold A Fischer M Ehlers JP. Fallbasiertes, elektronisches Lernen und Prüfen in der Tiermedizin - Auf der Suche nach einer Alternative zu Multiple-Choice Prüfungen. Tierarztl Umsch. (2011) 66:261–8.

[63] Brightwell R Daniel J-H Stewart A. Evaluation: is an open book examination easier? Biosci Educ. (2015) 3:1–10. doi: 10.3108/beej.2004.03000004

[64] Heijne-Penninga M Kuks JB Schonrock-Adema J Snijders TA Cohen-Schotanus J. Open-book tests to complement assessment-programmes: analysis of open and closed-book tests. Adv Health Sci Educ Theory Pract. (2008) 13:263–73. doi: 10.1007/s10459-006-9038-y 17063381

[65] Sam AH Reid MD Amin A. High-stakes, remote-access, open-book examinations. Med Educ. (2020) 54:767–8. doi: 10.1111/medu.14247, PMID: 32421858 PMC7276865

[66] Choi JH Hickman KE Monahan A Schwarcz DB. Chatgpt goes to law school. J Legal Educ. (2022) 71:387–400. doi: 10.2139/ssrn.4335905

[67] Kung TH Cheatham M Medenilla A Sillos C De Leon L Elepano C. Performance of ChatGPT on USMLE: potential for AI-assisted medical education using large language models. PLOS Digit Health. (2023) 2:e0000198. doi: 10.1371/journal.pdig.0000198, PMID: 36812645 PMC9931230

[68] Jung LB Gudera JA Wiegand TLT Allmendinger S Dimitriadis K Koerte IK. Chatgpt passes German state examination in medicine with picture questions omitted. Dtsch Arztebl Int. (2023) 120:373–4. doi: 10.3238/arztebl.m2023.0113, PMID: 37530052 PMC10413971

[69] OpenAI. Gpt-4 technical report. (2023). Ithaca, NY, USA: arXiv.

[70] Terwiesch C. Would ChatGPT3 get a Wharton MBA? A prediction based on its performance in the operations management course. (2023). Available at: https://mackinstitute.wharton.upenn.edu/wp-content/uploads/2023/01/Christian-Terwiesch-Chat-GTP.pdf

[71] Friederichs H Friederichs WJ Marz M. Chatgpt in medical school: how successful is AI in progress testing? Med Educ. (2023) 28:2220920. doi: 10.1080/10872981.2023.2220920, PMID: 37307503 PMC10262795

[72] Susnjak T. ChatGPT: the end of online exam integrity? (2022). Ithaca, NY, USA: arXiv.

[73] Cotton DRE Cotton PA Shipway JR. Chatting and cheating: ensuring academic integrity in the era of ChatGPT. Innov Educ Teach Int. (2023) 61:228–39. doi: 10.1080/14703297.2023.2190148

[74] Oravec JA. Artificial intelligence implications for academic cheating: expanding the dimensions of responsible human-AI collaboration with ChatGPT. J Interact Learn Res. (2023) 34:213–37.

